
JOURNAL INFORMATION
==============================
NLM Title Abbreviation: Behav Neurol
Journal ID: Behav Neurol
NLM Journal ID: 8914585
Journal ID: BN

Behavioural Neurology

ISSN: 0953-4180
EISSN: 1875-8584
Publisher: Wiley

ARTICLE INFORMATION
==============================
PMCID: PMC5434416
PMID: 21422555
DOI: 10.3233/BEN-2010-0295
Article ID: 946315
Article version: 1
Subjects: Research Article

Cognitive Decline in Normal Ageing and Early Alzheimer’s Disease: A Continuous or Discontinuous Transition?

Spaan Pauline E. J. *
Dolan Conor V.
Department of Psychonomics Clinical Neuropsychology University of Amsterdam Roetersstraat 15 1018 BS Amsterdam The Netherlands
*Pauline E. J. Spaan: p.e.j.spaan@uva.nl
Print publication date: 2010
Electronic publication date: 2011 Mar 7
Volume: 23
Issue: 4
First page: 203
Last page: 206
Received 2011 Jan 31; Accepted 2011 Jan 31
Copyright: Copyright © 2010 Hindawi Publishing Corporation and the authors.
Copyright year: 2010
License: This is an open access article distributed under the Creative Commons Attribution License, which permits unrestricted use, distribution, and reproduction in any medium, provided the original work is properly cited.
License URL: https://creativecommons.org/licenses/by/3.0/

==============================
Behavioural Neurology 23 (2010) 203–206
DOI 10.3233/BEN-2010-0295
IOS Press

### Cognitive decline in normal ageing and early Alzheimer's disease: A continuous or discontinuous transition?

Pauline E.J. Spaan* and Conor V. Dolan
*Department of Psychonomics, Clinical Neuropsychology, University of Amsterdam, Roetersstraat 15, 1018 BS Amsterdam, The Netherlands*

### 1. Objective / introduction

A longstanding debate in dementia research (e.g. [2]) has been whether normal ageing and Alzheimer's Disease (AD) are extremes that lie along the same continuum (continuity view), or whether AD is categorically different from normal ageing (discontinuity view). As age is the one and only promoting factor for developing AD, it is appropriate to examine its relationship to normal ageing. This question has become more and more relevant for clinical practice considering increased average life expectancy and, consequently, increased prevalence of dementia. Thus, does everyone who lives long enough eventually develop dementia or AD in particular? In other words, are cognitive changes at very old age *really* different from cognitive decline in (early) AD? If they are not, these changes would only differ in degree, at a quantitative level, which would be in favour of the continuity hypothesis. The discontinuity hypothesis, however, states that these changes not only differ in degree but also in kind, at a qualitative level.

Some studies report results that may be interpreted in favour of the continuity hypothesis (e.g. [1,4,8]), whereas other studies seem to adhere to the discontinuity hypothesis (e.g. [3,5,9]). This discrepancy may be explained by several methodological issues. First of all, the characteristics of the contrasted clinical groups may differ a lot. More specifically, the investigation of differences in performance between very healthy and very demented participants may cause a bias in favour of the discontinuity view. Secondly, reduced construct validity of the administered neuropsychological tests (i.e., one test often represents the influence of more than one cognitive process) complicates the detection of specific patterns of cognitive performance. Thirdly, in many studies only a few tests (i.e., measuring only one or a few cognitive functions) or even only cognitive screening tests were administered. This limits the possibility to derive a sufficiently broad cognitive profile from the results. Both the second and the third issue may lead to conclusions in favour of the continuity view. Finally, studies often differ in the adopted statistical approach. Commonly, the investigation is at the level of individual *tests* but conclusions are drawn at the latent level of cognitive *functions*, which disregards the examination of the interrelations between cognitive variables. We argue that only a latent variable approach testing for measurement equivalence is appropriate to investigate whether qualitative rather than quantitative differences in neuropsychological test performance exist between normal ageing and AD (e.g., Little, 1997).

We have attempted to overcome these methodological problems and tested the hypotheses of continuity versus discontinuity by means of a structural equation modeling approach using the data of a representative sample of early or preclinical AD patients and matched controls on a newly developed neuropsychological test battery.

### 2. Participants and methods

The sample of participants included a group of 43 AD and 37 amnestic Mild Cognitive Impairment pa-

*Corresponding author. E-mail: P.E.J.Spaan@uva.nl.

ISSN 0953-4180/10/$27.50 © 2010 – IOS Press and the authors. All rights reserved

Table 1

Fit indices of the invariance analyses across the two diagnostic groups (80 early or preclinical AD patients versus 80 healthy controls, matched for age, education, and gender)

| Model | Fit indices | | | | | | | $\chi^2$ difference test | | | |
|---|---|---|---|---|---|---|---|---|---|---|---|
| | $\chi^2$ | *df* | $\chi^2/df$ | CFI | NNFI | CAIC | RMSEA (90% CI) | Comp. to | $\Delta\chi^2$ | $\Delta$ *df* | *p* |
| M1: configural invariance | 285.37 | 218 | 1.31 | 0.962 | 0.952 | 819.99 | 0.063 (0.040–0.082): *Adequate fit* | | *baseline* | | |
| M2: measurement invariance – metric invariance | 360.39 | 230 | 1.57 | 0.935 | 0.923 | 822.10 | 0.085 (0.068–0.101): *Mediocre fit* | M1 | 75.02 | 12 | $< .001$ *Reduced fit* |
| M3: strong factorial invariance | 397.98 | 242 | 1.64 | 0.924 | 0.915 | 993.34 | 0.090 (0.074–0.106): *Mediocre fit* | M2 | 37.59 | 12 | $< .001$ *Reduced fit* |

*Note.* M1 = configural invariance: in this model, the configuration of factors (i.e., the factor structure containing the five factors: episodic memory; fluency; naming; processing speed; executive functioning) was identical for both groups, but the parameters (i.e., the magnitude of the *factor loadings*: the regression coefficients of the measurement (test) variables on the latent cognitive constructs or factors) were *free to vary* across the two groups.

M2 = measurement invariance – metric invariance: in this model, the *factor loadings* were *constrained to be identical* in both groups. (Note that this does not imply that the group means are identical.)

M3 = strong factorial invariance: this model specified that across both groups, the *factor loadings* and the *intercepts* in the regression of the measurement (test) variables on the (latent) factors were *equal*.

CFI = comparative fit index; NNFI = nonnormed fit index; CAIC = consistent Akaike information criterion; RMSEA = root-mean-square error of approximation; Comp. to = model compared to.

tients (of whom 21 had converted to AD at, on average, 1.3-year-follow-up; MMSE: $M$ = 24.8, *SD* = 2.5), and a group of 80 age-, education, and gender-matched non-demented controls (MMSE: $M$ = 28.9, *SD* = 1.0). Official diagnoses were made by a (blind) neurologist or geriatrician based on standard clinical procedures [7,10]. Exclusion criteria were: a history of (other) neurological or psychiatric disorders; use of psycho-active medication or substance abuse; another native language than Dutch; impaired vision interfering with test performance; missing values on more than two test measures.

These participants were administered a computerized battery of 17 test measures reflecting: (1) episodic memory, (2) fluency, (3) naming, (4) processing speed, and (5) executive functioning. Tests were constructed to measure the intended cognitive process as purely as possible, e.g., by minimizing the impact of short-term memory or executive control processes on episodic and semantic memory measures. To avoid large variance in reaction times on the measures of processing speed and executive functioning due to physical instead of cognitive limitations, no motor responses were required from participants. In addition, the battery included tests that each measured a different facet of memory or attention.

Multigroup Confirmatory Factor Analysis (LISREL 8.80) was performed to examine the invariance of the 5-factor neuropsychological model across the two groups (early AD patients versus controls).

### 3. Results

On all test measures, the patients performed significantly worse than the controls (paired $t$ tests: $p <$ 0.001). However, this is a purely *quantitative* approach and only focuses on the level of performance for each test separately. This does not provide information on whether also qualitative differences exist between normal ageing and early AD.

First, two independent confirmatory factor analyses determined that the 5-factor model mentioned above represented the best fitting factor structure of the test battery in both the early AD group ($\chi^2$ = 115, *df* = 109, RMSEA = .026) and in the control group ($\chi^2$ = 170, *df* = 109, RMSEA = 0.084).

Second, invariance analyses presented in Table 1 showed that the model had adequate configural invariance across the two groups, but neither metric nor strong factorial invariance ($p <$ 0.001). Thus, the pattern of factor loadings (i.e., the relations between the test variables and the latent cognitive constructs) is not similar between the patients and their controls. This suggests that the results are not compatible with the interpretation that the group differences are only quantitative; in addition, differences on the latent variables may be present.

### 4. Conclusion

The results indicate that the neuropsychological tests measure fundamentally different cognitive processes in normal ageing than in early AD. Qualitative rather than only quantitative differences in cognitive functioning seem to exist. These results provide evidence against the continuity hypothesis and in favour of the discontinuity hypothesis.

This outcome emphasizes the importance of developing tests that are able to detect the precise nature of the cognitive problems characteristic of early AD, up to very old age. This may be even more important than improving normative data or determining optimal cut-off scores of (existing) neuropsychological tests.

### References

[1] L. Bäckman, S. Jones, A.K. Berger, E.J. Laukka and B.J. Small, Cognitive impairment in preclinical Alzheimer's disease: A meta-analysis, *Neuropsychology* **19**(4) (2005), 520–531.

[2] G.F. Berrios, Dementia and aging since the nineteenth century, in: *Dementia and normal aging*, F.A. Huppert, C. Brayne and D.W. O'Connor, eds, Cambridge: Cambridge University Press, 1994, pp. 15–40.

[3] G.A. Carlesimo, M. Mauri, A.M.S. Graceffa, L. Fadda, A. Loasses, S. Lorusso and C. Caltagirone, Memory performances in young, elderly, and very old healthy individuals versus patients with Alzheimer's disease: Evidence for discontinuity between normal and pathological aging, *Journal of Clinical and Experimental Neuropsychology* **20**(1) (1998), 14–29.

[4] F.A. Huppert and C. Brayne, Wat is the relationship between dementia and normal aging? in: *Dementia and normal aging*, F.A. Huppert, C. Brayne and D.W. O'Connor, eds, Cambridge: Cambridge University Press, 1994, pp. 3–12.

[5] D.K. Johnson, M. Storandt, J.C. Morris and J.E. Galvin, Longitudinal study of the transition from healthy aging to Alzheimer Disease, *Archives of Neurology* **66**(10) (2009), 1254–1259.

[6] T.D. Little, Mean and covariance structures (MACS) analyses of cross-cultural data: Practical and theoretical issues, *Multivariate Behavioral Research* **32** (1997), 53–76.

[7] G. McKhann, D. Drachman, M. Folstein, R. Katzman, D. Price and E.M. Stadlan, Clinical diagnosis of Alzheimer's disease: Report of the NINCDS-ADRDA Work Group under the auspices of Department of Health and Human Services Task Force on Alzheimer's Disease, *Neurology* **34** (1984), 939–944.

[8] T.A. Salthouse and J.T. Becker, Independent effects of Alzheimer's disease on neuropsychological functioning, *Neuropsychology* **12**(2) (1998), 242–252.

[9] K.L. Siedlecki, L.S. Honig and Y. Stern, Exploring the structure of a neuropsychological battery across healthy elders and those with questionable dementia and Alzheimer's disease, *Neuropsychology* **22**(3) (2008), 400–411.

[10] B. Winblad, K. Palmer, M. Kivipelto, V. Jelic, L. Fratiglioni, L.-O. Wahlund et al., Mild cognitive impairment – Beyond controversies, towards a consensus: Report of the International Working Group on Mild Cognitive Impairment, *Journal of Internal Medicine* **256** (2004), 240–246.
